# Technology used in activity based therapy for individuals living with spinal cord injury across Canada

**DOI:** 10.1038/s41394-022-00558-y

**Published:** 2023-01-16

**Authors:** Cindy Gauthier, Kristen Walden, Hope Jervis-Rademeyer, Kristin E. Musselman, Anita Kaiser, Dalton L. Wolfe, Vanessa K. Noonan, Sarah J. Donkers

**Affiliations:** 1 Department of Physical Therapy, Faculty of Medicine, University of Toronto, Toronto, ON Canada; 2 KITE, Toronto Rehabilitation Institute –University Health Network, Toronto, ON Canada; 3 Praxis Spinal Cord Institute, Vancouver, BC Canada; 4 Rehabilitation Sciences Institute, Faculty of Medicine, University of Toronto, Toronto, ON Canada; 5 Parkwood Institute, Lawson Health Research Institute, London, ON Canada; 6 International Collaboration on Repair Discoveries, Vancouver, BC Canada; 7 School of Rehabilitation Science, College of Medicine, University of Saskatchewan, Saskatoon, SK Canada

**Keywords:** Rehabilitation, Rehabilitation

## Abstract

**Study design:**

Cross-sectional equipment inventory.

**Objectives:**

The objective of this study was to describe the equipment used in activity-based therapy (ABT) programs for individuals with spinal cord injury or disorder (SCI/D) across Canada.

**Settings:**

Publicly funded and private SCI/D care settings.

**Methods:**

A survey on equipment available for ABT for different therapeutic goals was answered by Canadian sites providing SCI/D rehabilitation. Information about the setting and type of client were also collected. The survey results were compiled into an inventory of the reported types and use of ABT related equipment, with equipment grouped into varying levels of technology. Descriptive statistics and qualitative descriptive analysis were used to answer the questions: (1) ‘who’ used the equipment, (2) ‘what’ types of equipment are used, (3) ‘why’ (i.e., for which therapeutic goals), and (4) ‘how’ it is used.

**Results:**

Twenty-two sites from eight Canadian provinces completed the survey. Reported equipment was classified into 5 categories *(from low to high-tech)*. Most equipment reported was used to train balance. The high-tech equipment reported as available, was mostly used for walking training and strengthening of the lower limbs. Low-tech equipment was reported as being used most frequently, while high-tech devices, although available, were reported as infrequently or rarely used.

**Conclusions:**

A large spectrum of equipment with varying levels of technology were reported as available, but were inconsistently used to provide ABT interventions across sites. In order to increase the clinical use of available equipment for ABT, education tools such as protocols regarding ABT principles and implementation are needed.

## Introduction

Following spinal cord injury or disorder (SCI/D), most individuals participate in a rehabilitation program to help regain function [1]. Rehabilitation interventions have commonly focused on training muscles above the level of injury and learning compensatory mechanisms [2]. In the past three decades, rehabilitation research in SCI/D has undergone a dramatic paradigm shift and now promotes the potential for neuroplasticity and subsequent recovery of function below the level of injury [3, 4]. An increased understanding of the mechanisms and potential for promoting neuroplasticity has led to the development of new approaches to rehabilitation such as activity-based therapy (ABT). ABT involves “repetitive neuromuscular activation below the level of spinal injury, typically achieved through intensive, task-specific movement practice” [3]. In line with the principles of activity-dependent neuroplasticity, ABT aims to both support the restoration of neurological impairments and decrease the risk of secondary complications associated with SCI/D [5]. As such, ABT initiatives should be of high training ‘dosage’ including volume, exercise intensity, and challenge level (e.g., frequency and duration of sessions, number of movement repetitions in a given time period, cardiovascular workload achieved and rate of perceived exertion) [6, 7].

An ABT Summit was held in 2019 to create a Canadian ABT Strategy for SCI/D [8]. About 40 participants including individuals with lived experience, clinicians, healthcare administrators, researchers and health policy experts attended the meeting. This Summit resulted in establishing five priorities to advance ABT research and care in Canada over the next 5 years. One of which was to identify current ABT activities across the continuum of care. Part of this priority was to capture a snapshot of current clinical care with regards to equipment use in ABT interventions across Canada for individuals living with SCI/D [8].

A variety of equipment and technologies are now available to facilitate the delivery of ABT. A recent environmental scan demonstrated that roughly two thirds of ongoing clinical trials in SCI/D rehabilitation involve the use of technology, such as electrical stimulation, robotic devices, virtual reality, or combinations of these technologies [3]. Despite the high uptake of technology in SCI/D rehabilitation-related research, the uptake in clinical practice is suspected to be much less. In fact, little is known about the actual clinical use of equipment and technology to deliver ABT to individuals living with SCI/D.

To begin to address this gap, the objective of this exploratory study was to identify and describe the current use of equipment in clinical ABT interventions across Canada. Specifically, we wished to explore what equipment occupational therapists (OT), physical therapists (PT) and other rehabilitation professionals reported using to provide ABT across the continuum of care. Moreover, we wanted to know how frequently the identified equipment was used, and for which functional goals and type of clients.

## Methods

### Development of the ABT survey

A survey was developed in collaboration with attendees of a 2019 ABT Summit [8] and the SCI Knowledge Mobilization Network [9]. The survey was piloted at a single rehabilitation centre to seek clarity on usability. Additional changes were made based on feedback before distributing. Ethical approval for this study was obtained by Praxis Spinal Cord Institute from the Veritas Independent Review Board.

The survey asked respondents to complete an Excel spreadsheet outlining the types of equipment available at their site to provide ABT according to common clinical goals (see Appendix A). The setting for use, type of client, and frequency of use were also collected.

### Survey respondents

Any site in Canada that had therapists who self-identified as using ABT interventions as part of their rehabilitation approach for individuals with SCI/D was eligible to participate. Publicly funded acute care and rehabilitation sites were recruited through a network of hospitals via the Rick Hansen Spinal Cord Injury Registry (RHSCIR) [10]. Non-publicly funded centres in the community were identified through the Canadian ABT Community of Practice [11] and by an Internet search for centres offering ABT in Canada. For the acute care and rehabilitation hospitals, recruitment targeted a site representative PT and OT, while at private practice clinics, it targeted one or two employees, including managers or clinic owners (as not all community sites have PTs and/or OTs).

### Survey completion

Data collection occurred from July 2019 to March 2020. During that time, an email was sent to participants which included the working definition being used for ABT [3] as well as the ABT survey for review. Participants were asked to complete the survey electronically based on information representative of their respective facility (hospital/clinic) and were provided researchers’ contact information in case of questions during completion. They were later provided with a copy of their survey results to review and validate. The final version of the survey was used to create an equipment inventory for data analysis.

### Analysis of the survey data

The equipment inventory allowed the researchers to answer the following questions: (1) Who? (what types of centres, therapists and clients), (2) What? (what type of equipment and level of sophistication (low to high-technology), (3) Why? (for which goals) and (4) How? (frequency of use, protocols, etc.) (details outlined below). Results were analyzed using descriptive analysis. Three researchers (CG, KW and SJD) analyzed the data and each question was addressed separately by two authors in a random order. The three authors (CG, KW, SJD) then shared their analyses for further discussion and consensus on equipment use for ABT.

#### Who?

The sites were categorized by the type of setting i.e., acute care hospital, rehabilitation centre or non-publicly funded centre in the community. The types of therapists were categorized by inpatient or outpatient PT or OT and rehabilitation assistant/kinesiologist/exercise therapist. Type of client was collected via a free text field, where the sites were asked to identify the American Spinal Injury Association Impairment Scale (AIS) [12], level of completeness (i.e., incomplete vs complete) and/or the body area impacted (i.e., tetraplegia or paraplegia).

#### What?

The types of equipment reported by participating sites were categorized based on their level of technological sophistication with regards to providing ABT [13, 14]:

##### Low-tech

Passive pieces of equipment (e.g., parallel bars, free weights, spin bike).

##### Low-medium tech

Motorized component that is not supporting movement action (e.g., electric lift or standing frame, tilt tables, walking sling attached to overhead track/lift).

##### Medium-tech

Motorized component that is supporting movement but does not directly facilitate the movement (e.g., a regular treadmill or an ergometer).

##### Medium-high tech

Motorized component that is directly facilitating the movement (e.g., an ergometer with active-assisted mode).

##### High-tech

Computer-based component or invasive energy (e.g., electrical stimulation) used to facilitate the movement (e.g., functional electrical stimulation [FES] devices, robotic devices).

There was also an ‘other’ category that included equipment difficult for researchers to interpret how it was being used to directly support ABT.

Some pieces of equipment could be considered high-tech in terms of their mechanical design, but the component or way it was actually used for the ABT intervention reflected lower tech. For example, an electronic plinth could be considered low-medium tech, but the technical part (i.e., the electronic part) is not used in ABT interventions since it cannot be used to directly stimulate or support movement of the affected limbs. In these cases, equipment was categorized based on their use specific to providing ABT interventions. Sites may have reported a brand while others reported the general type of equipment (e.g., functional electrical bike). Hence, the pieces of equipment were regrouped into categories of use or device type.

#### Why?

Each site was asked to list the equipment available for seven general functional goals (i.e., sitting and standing balance, walking, strengthening of the upper limb, strengthening of the lower limb, wheelchair propulsion, upper limb function [functional tasks] and cardiovascular, fitness and general wellness). In the category ‘other’, participants were asked to indicate any other equipment and technologies that were used for ABT interventions to train goals other than the ones outlined.

#### How?

For each specific functional goal, sites were asked to identify the extent of clinical (vs. research) use of the equipment in the client group they deemed appropriate, expressed in percentage ranges (Most of the time (≥80%), Frequently (60–79%), Sometimes (41–59%), Occasionally (21–40%), Rarely (1–20%), Never (0%), Planning to use but haven’t started and N/A – Research use only. Participants could also report any protocols or guidelines followed for use of a specific piece of equipment and add comments.

## Results

### Who?

In total, twenty-two different sites including two acute hospitals, eleven rehabilitation centres and nine non-publicly funded centres (private practice) in the community completed the inventory survey. Sites were located in Alberta (*n* = 4), British Columbia (*n* = 2), Ontario (*n* = 8), Quebec (*n* = 3), Manitoba (*n* = 1), New Brunswick (*n* = 1), Nova Scotia (*n* = 1), and Saskatchewan (*n* = 2). Private practice clinics included both not-for-profit organizations (*n* = 4) and for-profit-organizations (*n* = 5), with most having a physical location (e.g., gym, clinics or home-based clinic) (*n* = 8) and one providing home services (*n* = 1).

Publicly funded settings (*n* = 13) all reported that both PT and OT provided ABT, with nine of the thirteen sites also reporting rehabilitation assistants and kinesiologists being involved. Five out of the nine non-publicly funded centres have PT providing ABT, three have OT and six have kinesiologists or trainers. Of those six non-publicly funded centres, three reported only having kinesiologists or trainers.

Across the seven functional goals, all sites reported providing therapy and using equipment to individuals with all levels and severity of injury. However, sites varied in the types of interventions and equipment offered to different clients. Some functional goals were reported to be addressed only with specific clients.

### What?

See Table 1 for equipment and technologies grouped and categorized by level of sophistication.

**Table 1:** Number of sites that reported each type of equipment grouped by technology level.

		Acute *n* = 2	Rehab *n* = 11	Community *n* = 9	Total across sites *n* = 22
Low Tech	Assistive pulleys, slings, + springs (overbed mounted)	1	4	2	**7**
	Bodysuits (therasuit, upsee)	0	0	2	**2**
	Bungee Mobility trainer (Neurogym Tech)	0	2	1	**3**
	Cardio machines (vitaglide, spin bike)	0	0	1	**1**
	Environmental set up (ramps, curbs, wheelchair skills lab, car transfers)	1	3	0	**4**
	Gait aids (cane, crutches, poles, platform walkers, gutter walkers, basic walkers)	1	4	4	**9**
	Hydrotherapy pool	0	4	1	**5**
	Manual wheelchairs	0	0	0	**0**
	Parallel bars	1	2	2	**5**
	Plinths (standard, hi-low)	1	2	3	**6**
	Props general (blocks, cones, etc.)	1	2	2	**5**
	Prop for facilitation of UL movement (skateboard, roller, balls, mobile arm support)	1	5	2	**8**
	Props for functional activities (toothbrushes/combs/writing utensils/aids)	1	3	1	**5**
	Resistance training free weights (therabands, hand weights, ankle weights)	1	5	3	**9**
	Resistance/fitness training machines:	0	0	0	**0**
	UL (uppertone gym, vitaglide, Rickshaw rehab exercise)	1	5	3	**9**
	LL (smith machine, leg press, leg curl, squat rack)	0	3	3	**6**
	Combo (weighted pulleys, bowflex, combo twist, total gym, reformer, keiser frame)	1	7	6	**14**
	Sit to stand trainer	0	2	0	**2**
	Splints/braces	0	3	1	**4**
	Standing frames/suspension support (non-motorized)	1	6	6	**13**
	Surface modifier for balance (foam, balance board, bosu, swiss ball, incline wedge)	1	1	3	**5**
	Suspension exercise trainer (TRX)	0	0	1	**1**
	**Total for Low Tech**	**13**	**63**	**47**	**123**
Low-Med	Electric/assistive lift standing frames (e.g. Rifton Tram)	1	7	1	**9**
	Electronic mobile arm support (Kinova)	0	1	0	**1**
	Tilt tables (including circo-lectric bed)	1	4	1	**6**
	Walking sling attached to overhead track/lift	2	6	0	**8**
	**Total for Low-Med Tech**	**4**	**18**	**2**	**24**
Med Tech	Elliptical	0	2	0	**2**
	Exercise bike (upright)	0	3	2	**5**
	Exercise bike (recumbent)	0	2	2	**4**
	Ergometer UL (that don’t include an active assist e.g. motomed, SCIFit)	2	9	5	**16**
	Ergometer LL	1	3	3	**7**
	FEPS (flexion, extension, pronation, supination, resistance trainer)	0	1	0	**1**
	Mobile electronic bodyweight and postural control support frames (Arjo, Litegait, Rifton)	0	6	5	**11**
	Recumbent stepper (Nustep)	0	7	3	**10**
	Resistance trainer with technology (baltimore equipment, HUR ab/ad trainer)	0	1	0	**1**
	Stepper/stair climber	0	1	1	**2**
	Racing wheelchair instrumented roller	0	0	3	**3**
	Rower	0	0	2	**2**
	Treadmill	0	6	2	**8**
	Wheelchair treadmill	0	1	0	**1**
	Vibration plate	0	0	4	**4**
	**Total Med Tech**	**3**	**42**	**32**	**22**
Med-High	Bodyweight support elliptical trainer (Madonna ICare Kintron)	0	2	1	**3**
	Bodyweight support treadmill trainer (e.g. woodway, therastride)	0	7	0	**7**
	Ergometers that do include an active assist (non-FES) (biodex)	0	2	1	**3**
	Power add-ons to manual wheelchair	0	2	0	**2**
	Standing feature on powered wheelchair	0	1	0	**1**
	Split belt treadmill	0	1	0	**1**
	**Total for Med-High Tech**	**0**	**15**	**2**	**17**
High Tech	Balance trainers (biosway, balance master, visual field stimulator, HUR balance lab)	0	4	1	**5**
	Computerized upper limb training devices (SaeboReJoyce, Armeo, Amadeo)	0	4	3	**7**
	Exoskeleton (Ekso)	0	6	1	**7**
	FES eliptical	0	1	1	**2**
	FES LL bike (RT-200, RT-300,)	1	8	5	**14**
	FES UL bike (RT-200, RT-300)	0	5	2	**7**
	FES orthotic (bioness, walkaid, odstock)	0	9	4	**13**
	FES rower	0	1	0	**1**
	FES stepper (RTI-600)	0	2	0	**2**
	FES machines for limbs (twinstim, myndmove, EMPI, Neurocare)	0	11	8	**19**
	FES for sit to stand and trunk (Xcite)	0	3	0	**3**
	Robotic gait system trainer (Lokomat)	0	2	1	**3**
	Robotic hippotherapy	0	0	1	**1**
	**Total for High Tech**	**1**	**56**	**27**	**84**

The count represents the number of sites that reported having the type of equipment, not the number of devices or brands.

The median value and interquartile range (IQR) for the number of equipment types based on level of technology are included in Table 2. Acute sites reported having mostly low-tech pieces of equipment and almost no medium-high or high-tech equipment. Rehabilitation sites reported mostly low- and high-tech equipment and fewer in the categories in between. Community sites reported a similar number of equipment across the low-, medium- and high-tech categories.

**Table 2:** Number of different types of equipment by technological sophistication per phase of care (Median (IQR)).

		Level of technological sophistication				
		Low	Low-medium	Medium	Medium-high	High
Setting	Acute	6.5 (3.25-9.75)	2 (1.5-2.5)	1.5 (1.25-1.75)	0 (0-0)	0.5 (0.25-0.75)
	Rehab	5.0 (3.5-6.5)	2 (1-2)	3 (3-5)	1 (1-2)	5 (4-6)
	Community	4 (4-8)	0 (0-0)	4 (1-5)	0 (0-0)	3 (2-5)

Overall, the most reported piece of equipment was FES devices including handheld devices. However, these were not reported by the acute sites (Acute = 0; Rehab = 11; Community = 8). Non-assisted ergometer for upper limb was the second most reported piece of equipment and was reported by most sites in each setting (Acute = 2; Rehab = 9; Community = 5).

The reported equipment that was classified into the ‘other’ category (i.e., no clear direct role in performing ABT, but perhaps augments assessment or feedback on performance of ABT) included: 3D printer; ultrasound; wax bath; vestibular camera; pressure sensor mat and/or walkway; force plate; inertial sensor; smart board/table; interactive light board (e.g., dynavision); iPad; video game (e.g., Wii fit); virtual reality, and non-invasive cranial stimulation (e.g., transcranial magnetic stimulation). The majority of this equipment was reported by a single site, but all of the equipment in the ‘other’ category was reported by less than 3 sites.

### Why?

**Fig. 1:**
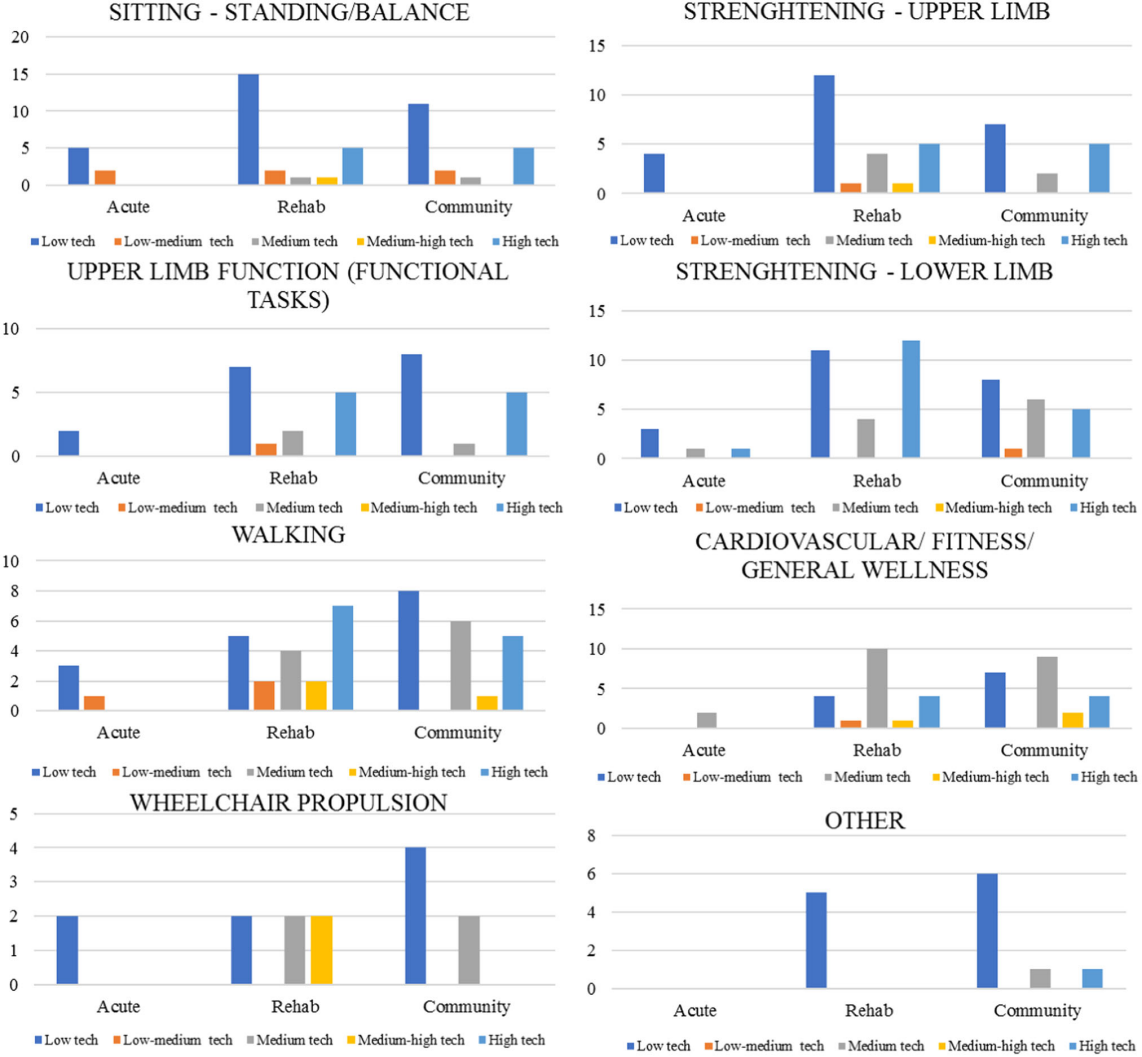
Frequencies of equipment types across settings based on reported goal. Number of types of equipment reported by the acute care hospitals, rehabilitation centres, or non-publicly funded centres in the community in each level of technology for each therapeutic goal.

Sites reported the equipment used for each functional goal. Frequencies for equipment type were grouped by functional goals (Fig. 1).

In total, the greatest variety of equipment was used to train sitting and standing balance (*n* = 60, 18.1% of the total) across sites and across technological sophistication categories. For this functional goal, all settings were using more low-tech pieces of equipment. However, rehabilitation and community settings also reported using a few high-tech devices and equipment classified in the ‘other’ category (especially video games, instrumented surfaces, virtual reality, and pressure sensors). For all functional goals, most of the equipment reported was low-tech (range 32.7–51.7%) except for the cardiovascular/fitness/general wellness goal, for which most equipment was considered medium-tech (46.7% of this goal) across all settings. For equipment reported across all goals, 41.9% was low-tech followed by 20.8% high-tech and 17.8% medium-tech.

In the high-tech category, the equipment was mostly used for strengthening of the lower limbs (26.1%) followed by walking training (17.4%). In this sophistication category, many devices using electrical stimulation were reported along with robotic gait trainers such as exoskeletons and the Lokomat. The goal that required the least amount of equipment was wheelchair propulsion (5.1% of the total of all devices) followed by the “Other” goals’ category (7.5% of the total).

### How?

In the acute and rehabilitation settings, most of the low-tech pieces of equipment were reported as being used more than 60% of the time (frequently or most of the time), while this same type of equipment was often reported to be used less than 60% of the time by community sites. For all the other levels of sophistication, the equipment was reported to be mostly used less than 60% of the time by all sites. There was an exception for the acute sites where one site reported to use medium tech (i.e. UE ergometer) frequently while the other site used it rarely.

For the most part, high-use was reported for the low-tech equipment. Notably however, standing assist devices, like standing frames and sit-to-stand trainers were reportedly used less than 60% of the time, despite high availability. With the exception of handheld FES devices, the medium- to high-tech pieces of equipment were used less than 60% of the time and some pieces in this category reported as available were never used (medium tech, *n* = 7; high tech, *n* = 7). Only devices in the high-tech category were reported as ‘used for research only’ or not being used yet but planning to be used in the future. Devices with this type of planned use were only reported by two rehabilitation centres.

## Discussion

This exploratory study aimed to identify the current use of equipment for ABT in different SCI/D care settings across Canada. Many pieces of equipment were reported, to assist with presenting results, equipment was grouped into types and categorized by level of technological sophistication. The majority of reported equipment fell into the low-tech followed by the high-tech category. The type of equipment used differed between therapeutic goals, with goals like walking using more sophisticated devices while goals like balance using mostly low-tech equipment. Differences in the reported type of equipment and its use were also noted between the types of care settings. Acute sites reported a majority of low-tech equipment while rehabilitation sites reported a larger number of high-tech devices. Sites in the community reported similar numbers of equipment in low-, medium and high-tech sophistication categories. It was difficult to identify patterns of use with regards to type and severity of SCI/D as all sites reported using equipment with all levels and severity of injury, and sites varied in the therapeutic goals they used types of equipment with for different clients.

### Comparing across settings: acute vs. rehabilitation vs. community

Therapeutic goals evolve and change across the continuum of care following SCI/D [15]. This evolution was illustrated in the use of ABT equipment for specific therapeutic goals in the different care settings. In acute settings, the primary ABT goal using equipment was improving sitting and standing balance, followed by strengthening of the lower and upper limbs. In this acute phase, individuals are often medically unstable and thus the functional goals that are achievable are likely limited. The length of stay in Canadian acute care settings is also on average shorter (i.e., 37.8 ± 58.6 days) than that of rehabilitation centres (i.e., 99.2 ± 77.1 days) [16]. The objective in an acute setting is primarily to medically stabilize individuals while initiating the process of rehabilitation and preparing them for a more intensive functional rehabilitation program offered by specialized SCI/D rehabilitations centres [17].

In rehabilitation centers, sitting and standing balance were also the functional goals with the most types of equipment reported, followed by lower limb strengthening, then walking, and upper limb strengthening. These results align with the most commonly reported rehabilitation goals set by patients and their multidisciplinary rehabilitation team; namely increasing mobility and participation in activities of daily living [18, 19]. Balance, strength and gait training are all prerequisites for mobility and activities of daily living. Only 4% of the equipment reported was for training of wheelchair propulsion. This percentage might not reflect the importance of this goal in the rehabilitation process as it can be achieved using minimal equipment.

In the community, devices were most commonly reported for the functional goals of improving sitting and standing balance, followed by cardiovascular fitness and general wellbeing, then walking, and lower limb strengthening. Some goals such as balance, wheelchair skills, and walking have been reported to decrease in importance throughout the years following the SCI/D while strengthening, fitness, and transfer skills increase [20]. Since balance is directly linked with safety and is one of the main concerns after a SCI/D [21, 22] it is understandable that the sites across all settings reported more pieces of equipment for this functional goal.

Across all settings, only a few pieces of equipment were reported to train wheelchair propulsion and they were directly related to the wheelchair, its components, and aspects of the environment. A few sites reported having rollers or a treadmill for wheelchairs available but reported their use to be rare. Since one of the main ABT principles is to target activation below the level of the SCI/D, for lower levels of injury it is possible that wheelchair propulsion was not considered an ABT activity. Additionally, as this functional goal might be easier to train independently by individuals at home, other functional goals requiring the assistance of the therapist or more sophisticated equipment might have been favored during therapy time.

### Use of equipment and technology

Several types of equipment across all sophistication categories were reported in more than one functional goal. While it is common to use low-tech equipment like weights and general props (e.g. blocks, cones) in versatile ways for different goals, it is interesting to note that high-tech devices were used for many different functional goals too, especially electrical stimulation devices like FES bikes and handheld electrical stimulation devices. Research has reported a lack of clinical uptake for technology in neurorehabilitation due to barriers, including a lack of time to learn how to use it and the cost [3]. The results of this study are consistent with these findings, showing less availability of high-tech devices than lower-tech devices and infrequent use (i.e., less than 60% of the time) of the available high-tech devices. However, considering these devices can be used to train multiple goals, and in some situations at the same time [23], it could be time efficient to use them after receiving sufficient training. Therapists have previously stated that being more comfortable and confident in using technology such as electrical stimulation would facilitate its use [23]. Interestingly, the middle three categories (low-med, med, med-high tech) were reported to be used less than 60% of the time across all sites. Knowledge transfer and learning activities, especially hands-on training, should be made available regularly to improve clinical use [23], and a better understanding is needed to help identify reasons beyond knowledge gaps for this discrepancy between availability and use. It is likely that unique strategies will be needed to address barriers based on the care context [24–26]. Some clinicians have reported that ABT activities vary widely and that they apply creativity in using the equipment in an unconventional way in order to apply the ABT principles [26]. Learning collaboratives, such as the Canadian ABT Community of Practice, represent great opportunities for knowledge sharing activities and to provide clinicians with the tools to increase their use of ABT and its associated equipment [27].

When analyzing the list of equipment reported by the sites, a discrepancy was noticed between the definition of ABT and its perceived understanding by the sites. Indeed, some of the equipment types reported did not seem to support the principles of ABT (e.g., the ‘other’ category), such as a large number of repetitions, stimulation below the level of injury and facilitating task specific movements [3]. However, fully exploring this discrepancy was beyond the scope of this study.

A recent qualitative research study conducted by Cheung et al. [26]. highlighted the large variety of ABT interventions as well as a gap in knowledge about ABT principles and their applications. To move the practice and science of ABT forward, a better understanding of its application is needed. The development and dissemination of protocols or guidelines to help share how equipment can be used to support and apply the principles of ABT would be beneficial. Moreover, it is not known how equipment dependent ABT needs to be. Some sites reported using very little equipment while describing interventions that followed many ABT principles [26]. The high cost of some types of equipment and their rapid evolution may be a potential barrier to explain why some sites do not have access to many types of high-tech equipment. This inventory could be used to help sites decide which type of devices are more valuable to purchase in their setting.

### Limitations

The results of this exploratory study should be interpreted with caution since a complete listing of available equipment along with specific uses may not have been reported by the sites. Some survey questions were not answered by all sites. There were also discrepancies in the way the equipment was presented between sites. Also, it was difficult to know exactly how a piece of equipment reported was used to provide ABT in order to classify it in the appropriate category. Most of the sites who participated in this survey were recruited via groups that were aware and involved in ABT development. Therefore, there could have been a selection bias and less specialized rehabilitation centres could be underrepresented.

### Future studies

Since the survey in this study was not designed to capture the ways in which clinicians were using reported equipment to provide ABT interventions, the addition of that information would be very useful. A better understanding of the discrepancy between equipment availability and use could help identify effective strategies for increasing integration of available equipment into routine practice. Further, investigating how therapists are applying the principles of ABT without using equipment should be explored. There was limited information on dosage provided by sites. Future studies are needed to better understand the use of some pieces of equipment to meet the key principles of ABT and target neurorecovery below the level of lesion. The data gathered in this study may help standardize terminology in order to enhance future initiatives and to better understand the nature of ABT-related practice.

## Conclusion

Equipment and its use to provide ABT interventions vary greatly in SCI/D care settings in Canada. The equipment identified represents a spectrum of technological sophistication (e.g. going from balls and weights to exoskeletons and FES). Despite the large number of devices reported, the results of this study demonstrated the gaps between available pieces of equipment and their actual use. This highlights the need for more education about ABT, including its core principles, associated equipment, and feasible usage protocols.

## Supplementary information

Appendix A

## Data Availability

The datasets analyzed during the current study are available from the authors or the Praxis Spinal Cord Institute on reasonable request.
