# Supplementary material for: Technology used in activity based therapy for individuals living with spinal cord injury across Canada
Source: Spinal Cord Ser Cases. 2023 Jan 16;9:1. doi: 10.1038/s41394-022-00558-y (PMC9842763; doi:10.1038/s41394-022-00558-y)
Supplement: Supplementary file 1 — Appendix A [file 41394_2022_558_MOESM1_ESM.pdf]

1) Please include both what is currently used in practice (including inpatients and outpatients) at your facility for clients with SCI as well as any equipment that therapists have access to (a device can be used in more than 1 category)

					CURRENT WORK/IMPLEMENTATION						
**Type of Therapy**	**Device (if applicable)**	**Research Use**	**Research - Type of client (e.g. AIS; incomplete/complete; tetraplegia/paraplegia)**	**Clinical Use - Inpatients**	**Inpatient - Type of client (e.g. AIS; incomplete/complete; tetraplegia/paraplegia)**	**Clinical Use - Outpatients**	**Outpatient - Type of client (e.g. AIS; incomplete/complete; tetraplegia/paraplegia)**	**Extent of Clinical Use in Clinically Appropriate Patients (considering all therapists)**	**Results/ Evaluation/ Successes**	**Protocols (Guidelines for Use) - please attach/ share if possible**	**Comments**
**SITTING - STANDING/BALANCE**	*e.g. evolutive standing frame*	*Yes*	*AIS C/D*	*Yes*	*incomplete tetraplegia & paraplegia; AIS C/D*	*Yes*	*incomplete tetraplegia & paraplegia; AIS C/D*	*Frequently (60%-79%)*	*well used by all therapists*	*no*	
**WALKING**	*e.g. exoskeleton*	*Yes*	*AIS C/D*	*Future work planned*	*n/a*	*Future work planned*	*n/a*	*Planning to use but haven't started*		*research protocol*	
	e.g. body weight support	*Yes*	*AIS C/D*	*Yes*	*incomplete tetraplegia & paraplegia; AIS C/D*	*Yes*	*incomplete tetraplegia & paraplegia; AIS C/D*	*Occasionally (21%-40%)*	*used very occasionnally in clinic, the time spent for set up is a big obstacle*	*yes*	
**STRENGHTENING - UPPER LIMB**	*e.g. Fitness and strenghtening machines (i.e. pulleys, UE ergometer, etc.)*	*Yes*	*all clients with UE function*	*Yes*	*all clients with UE function*	*Yes*	*all clients with UE function and related goals*	*Most of the time (≥ 80%)*	*use of rehab assistant and strengthening classes helpful*	*no*	
	e.g. FES- hand	*Yes*	*incomplete tetraplegia*	*no*	*n/a*	*no*	*n/a*	*n/a - research use only*	*results of the research project were inconclusive for our clientele so no standard clinical use planned*	*research protocol*	
**STRENGHTENING - LOWER LIMB**	*e.g. nu-step*	*No*	*n/a*	*Yes*	*AIS C/D*	*Yes*	*AIS C/D*	*Frequently (60%-79%)*			
	e.g. RehaMove FES Cycling system	*Yes*	*AIS C/D*	*Yes*	*AIS C/D*	*Yes*	*AIS C/D*	*Occasionally (21%-40%)*	*kept in separate room from main treatment area, time required for set up, and therpiats comfort with use are obstacles*	*research protocol*	
**WHEELCHAIR PROPULSION**	*e.g. Racing wheelchair instrumen*	*Yes*	*Manual w/c users*	*Future work planned*	*n/a*	*Yes*	*manual w/c users with related goals*	*Sometimes (41%-59%)*	*newly introduced to outpatient use*	*research protocol*	
**UPPER LIMB FUNCTION (FUNCTIONAL TASKS)**	*e.g. Kinect*	*Yes*	*tetraplegia*	*Yes*	*tetraplegia*	*Yes*	*tetraplegia*	*Sometimes (41%-59%)*	*limited patient access outside therapy as device available in therapy gym only*		
	e.g. Jintronix	*Yes*	*tetraplegia*	*No*	*n/a*	*No*	*n/a*	*n/a - research use only*	*currently research use only*	*research protocol*	
**CARDIOVASCULAR/ FITNESS/ GENERAL WELLNESS**	*e.g. Racing wheelchair instrumented roller*		*Manual w/c users*	*Future work planned*	*n/a*	*Yes*	*manual w/c users with related goals*	*Sometimes (41%-59%)*	*newly introduced to outpatient use*	*research protocol*	
**OTHER**	*e.g. magnetic transcranial stimula*	*Yes*		*Future work planned*	*n/a*	*Future work planned*	*n/a*	*n/a - research use only*		*research protocol*	
	e.g. virtual reality	*Yes*		*Future work planned*	*n/a*	*Future work planned*	*n/a*	*n/a - research use only*		*research protocol*	

2) Please outline the typical amount of therapy provided at your facility (please add rows as needed for each therapy type)

**Therapy Type**	**Avg. # sessions per week/client**	**Avg. length of session (minutes)**	**Type of client (e.g. AIS; incomplete/complete; tetraplegia/paraplegia)**
Inpatient PT			
Outpatient PT			
Inpatient OT			
Outpatient OT			
Rehab Assistant /Kinesiologist/ Other			

Group Classes Available (List below):			
e.g. UE fine movement/hand class	*2*	*60*	*tetraplegia*

3) Do you know of any community resources where there are opportunities to engage in ABT (please list)?
